# 31st Brazilian Online Society for Virology (SBV) 2020 Annual Meeting

**DOI:** 10.3390/v13030414

**Published:** 2021-03-05

**Authors:** Luciana Barros de Arruda, Fabrício Souza Campos, Jônatas Santos Abrahão, Flávio Guimarães da Fonseca, João Pessoa Araújo Junior, Fernando Rosado Spilki

**Affiliations:** 1 Laboratório de Genética e Imunologia das Infecções Virais, Depto de Virologia, Instituto de Microbiologia Paulo de Góes, Universidade Federal do Rio de Janeiro (UFRJ), Rio de Janeiro, RJ 21941-902, Brazil; 2 Bioinformatics and Biotechnology Laboratory, Campus de Gurupi, Universidade Federal do Tocantins (UFT), Gurupi, TO 77410-570, Brazil; camposvet@gmail.com; 3 Department of Microbiology, Universidade Federal de Minas Gerais (UFMG), Belo Horizonte, MT 31270-901, Brazil; jonatas.abrahao@gmail.com (J.S.A.); fdafonseca@icb.ufmg.br (F.G.d.F.); 4 Institute of Biotechnology, Universidade Estadual Paulista (UNESP), Botucatu, SP 18607-440, Brazil; joao.pessoa@unesp.br; 5 One Health Laboratory, Federação de Estabelecimentos de Ensino Superior em Novo Hamburgo (FEEVALE), Novo Hamburgo, RS 93525-075, Brazil

**Keywords:** human virology, veterinary virology, viral pathogenesis, antivirals, viral epidemiology, viral diseases, COVID-19

## Abstract

The year 2020 was profoundly marked by the emergence and spread of SARS-CoV-2, causing COVID-19, which represents the greatest pandemic of the 21st century until now, and a major challenge for virologists in the scientific and medical communities. Increased numbers of SARS-CoV-2 infection all over the world imposed social and travel restrictions, including avoidance of face-to-face scientific meetings. Therefore, for the first time in history, the 2020 edition of the Brazilian Society of Virology (SBV) congress was totally online. Despite the challenge of the new format, the Brazilian society board and collaborators were successful in virtually congregating more than 921 attendees, which was the greatest SBV participant number ever reached. Seminal talks from prominent national and international researchers were presented every night, during a week, and included discussions about environmental, basic, animal, human, plant and invertebrate virology. A special roundtable debated exclusively new data and perspectives regarding COVID-19 by some of the greatest Brazilian virologists. Women scientists were very well represented in another special roundtable called “Young Women Inspiring Research”, which was one of the most viewed and commented section during the meeting, given the extraordinary quality of the presented work. Finally, SBV offered the Helio Gelli Pereira award for one graduate and one undergraduate student, which has also been a fruitful collaboration between the society and *Viruses* journal. The annual SBV meeting has, therefore, reached its goals to inspire young scientists, stimulate high-quality scientific discussion and to encourage global collaboration between virologists.

## 1. Introduction

The Brazilian Society of Virology (SBV) represents and congregates professionals and students enrolled in different areas of the virology field, aiming to promote human, animal and plant health; stimulate the development of alternative technologies; preserve the environment and promote sustainable development; increment the knowledge regarding viral wealth and diversity; encourage collaboration between national and international scientific institutions; and disseminate science to the general public.

For the past 31 years, the society promoted the Brazilian Congress of Virology and Mercosur Virology Meeting, which was usually held in a different city each year, aiming to encourage and stimulate a collaborative network among researchers from different regions of Brazil, and among Brazilians and international researchers. In 2020, The 31st SBV Meeting was compelled to happen entirely online due to the rapid and severe spread of the SARS-COV-2 infection and COVID-19 pandemic, which imposed new social-distance rules.

Despite the challenge of a new format, this meeting had the highest number of attendees, seeking to discuss high-quality science and recent advances in the overall virology fields, especially regarding COVID-19. Seminal presentations by researchers from different countries included recent knowledge and unpublished data regarding host response and immune metabolism during virus infection, environmental virology, host range evolution, embracing human-animal interface and virus emergence, metagenomics and high-throughput analysis to investigate virus evolution and surveillance, and antiviral vaccines. Remarkably, discussions regarding various aspects of COVID-19 were present all through the meeting, reflecting the engagement of SBV and all collaborators and participants in carrying out the responsibility to confront and understand major features concerning the pandemic.

Importantly, about 750 students participated in the event and could send abstracts for divulgation in the society webpage; some of those students were selected to share their work in oral sessions, having the opportunity to discuss with senior and renowned researchers in their field.

Therefore, the SBV annual meeting fulfilled the major goal of a scientific society in promoting high-quality science discussion and stimulating the development and increase of well-qualified human resources committed to the advancement of science.

## 2. The Scientific Program of the 31st SBV Annual Meeting

The scientific program of the 31st SBV Annual Meeting included seven plenary conferences, three roundtables and seven short presentation sections, presented by a total of 53 speakers. Those included 15 senior scientists from Brazil (10), Spain (1) and the United States (4), besides 37 young researchers and students, who were previously selected based on their submitted abstracts. In addition, we had the help of 21 chairs who conducted those conferences, the Helio Gelli Pereira (HGP) award, and the SBV general assembly.

Conferences were based on recent development, including unpublished data, regarding basic, environmental, veterinary, human, plant and invertebrate virology. Several aspects of COVID-19 were debated in various conferences, including virus biology, disease pathogenesis, immune response and vaccines, highlighting the one-health concept and the relevance of emerging viruses’ surveillance.

Undergraduates, graduate and recently graduated students could also apply for the HGP award, which is traditionally conferred by SBV to the best full article developed by a Brazilian student. Importantly, the 2020 SBV meeting had the greatest student participation in the history of the society, which reflects the continuous commitment of the SBV in promoting interactions among young and prominent researchers in the field, contributing to the development and consolidation of their scientific careers.

Among the speakers of the plenary conferences and roundtables, 11 were men and only four were women. In addition, the scientific committee comprised 12 men and nine women, emphasizing the necessity to improve female participation in SBV meetings as speakers. On the other hand, 59% (543/921) of the attendees were women, 40.9% (377/921) were men, and 0.1% (1/921) was transgender. These perspectives show that we must improve and recognize women’s participation in the most relevant activities in future meetings to realistically represent everyone involved in each virology field. To minimize this big difference, we made an exclusive Young Women Inspiring Research roundtable, in which two young female scientists could discuss their careers and recent scientific developments.

More information about the event may be found on the meeting website, https://www.even3.com.br/cbv2020/ (accessed on 27 February 2021) [1] and on the society website https://sbv.org.br/sbv/ (accessed on 27 February 2021) [2].

### 2.1. Attendants at the Meeting

**Figure 1:**
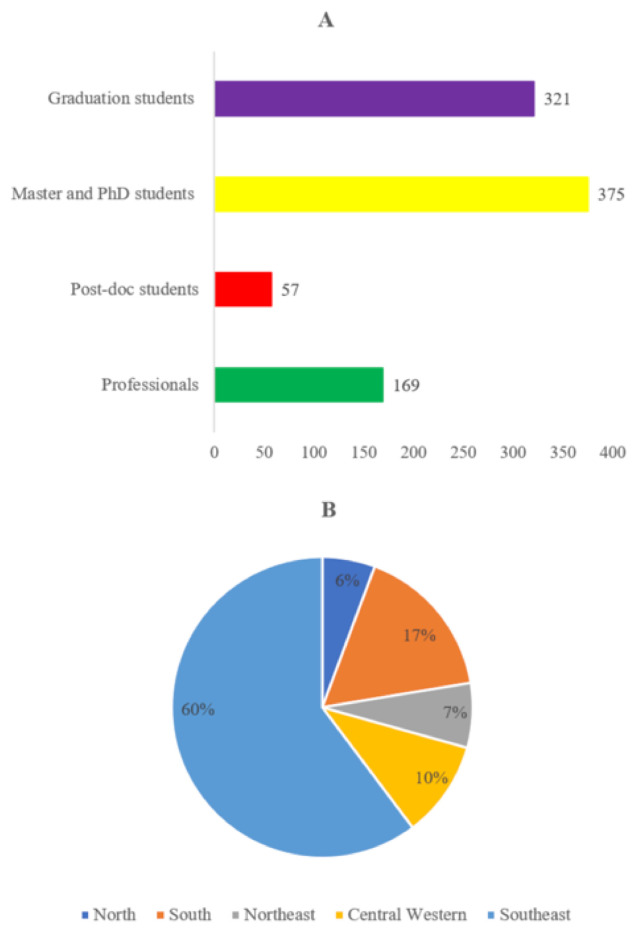
(**A**) Frequency of participants of the 31st Brazilian Society for Virology Congress and 15th Mercosul Virology Meeting by category and (**B**) their respective institution location in each country region.

The 31st SBV Annual Meeting had a record participation with 921 attendees, comprising professionals (18%), post-docs (6%), Masters and PhD students (41%), and graduated students (35%) (Figure 1A). This total included participants from the Southeast (majority), Central Western, South, North, and Northeast regions of Brazil (Figure 1B). 

### 2.2. Scientific Program

The global situation caused by the SARS-CoV-2 pandemic, including travelling and social restrictions, compelled all the scientific meetings, including the SBV annual congress, to happen entirely online. The event was held from 23–28 November 2020, with an average of two hours of activities every night, including conferences, roundtables, short and oral presentations, and the HGP award. The speakers were distributed over seven plenary conferences, three roundtables, and seven sections of short oral presentations performed by selected students and young researchers. Conferences covered different fields of virology, including the most recent advances in COVID-19 research regarding SARS-CoV-2 replication, epidemiology, and human-animal interface, in addition to host response and immunometabolism (Table 1). In 2020, SBV also conducted the election for the biennial directory board, which was held during the general assembly on the last day of the meeting.

### 2.3. Conference Speakers and Presentations

Dr. Robert E. Schwartz is an Assistant Professor of Medicine at the Sanford I. Weill Medical College of Cornell University and an Attending Physician at the New York–Presbyterian Hospital Cornell campus of Cornell University, New York City, NY, United States. With a large experience in gastroenterology, hepatology and metabolism, over the past year Dr. Schwartz has been studying different features of COVID-19, including immunometabolism, epigenetic alterations and the evolution of antibody responses, which were covered in his exceptional opening conference presentation entitled “The landscape of host responses and disease pathology in SARS-CoV-2 infection”.

Dr. Edson Elias Silva was a former president of SBV (2007–2008) and is a full researcher from Instituto Oswaldo Cruz, Fiocruz, at Rio de Janeiro, Brazil, where he coordinates the Enterovirus Laboratory of the Virology Department. He also works as a consultant for the Pan American Health Organization (PAHO) and WHO Laboratories within the Global Polio Eradication Program. Dr. Silva’s talk, “Environmental surveillance of enterovirus in support of global eradication activities,” pleased the audience with his long expertise in the field and stimulated discussions about the recent advances regarding polio eradicating policies.

Dr. Fernando Garcia-Arenal is a professor from the Center of Biotechnology and Plant Genomic, at Universidad Politécnica de Madrid, Spain, who presented the conference talk “Ecological complexity, host range evolution, and virus emergence”. Dr. Garcia-Arenal is a specialist in plant-virus interaction and co-evolution and in the investigation of host fitness functions that interfere and are modulated by virus infection, impacting the emergence of new viral diseases.

Dr. Pedro Manoel Mendes de Moraes Vieira is a professor of immunology at the department of genetics, microbiology and immunology, at the Institute of Biology, UNICAMP, SP, Brazil. He is also an affiliated member of the São Paulo State Academy of Sciences. Dr. Vieira has been dedicating his career to investigate immunology and metabolism, with a focus in obesity and diabetes. In the past year, his group has developed important studies about the interplay between glucose metabolism, inflammatory response, and SARS-CoV-2 infection, which were encompassed in his presentation, entitled “Targeting immunometabolism to understand COVID-19 disease”.

Dr. Diego Diel, who presented the fascinating conference talk “The human–animal interface of SARS-CoV-2”, is an associate professor at Cornell University College of Veterinary Medicine, NY, United States. His main research interest regards the identification and characterization of molecular mechanisms associated with host cell evasion by different animal viruses, which are relevant for disease pathogenesis.

Finally, we had two conferences from researchers associated with very important companies in the field, who presented the most recent advances of new generation sequencing (NGS) for different virus investigative goals. Fernando Rivadavia, a Technical Sales Specialist at Illumina (Miami, Florida, USA), spoke about “The role of new generation sequencing (NGS) in combating COVID-19”. Allan Munford, Customer Solution Manager at QIAGEN (São Paulo, Brazil), presented the talk “Unraveling the SARS-CoV-2 epidemiology by new generation sequencing (NGS)”.

### 2.4. Roundtables

Roundtable 1 comprised an update about COVID-19 in Brazil. Dr. Eurico de Arruda Neto (Faculdade de Medicina de Ribeirão Preto, Universidade de Sao Paulo -FMRP/USP, SP, Brazil) was a chair and speaker, and presented “A brief look at COVID-19 pathogenesis”. Dr. Jorge Kalil (Faculdade de Medicina, USP - FM/USP, SP, Brazil) lectured about “The worldwide effort to develop a safe and effective vaccine against COVID-19”. Dr. Renato Santana de Aguiar (Universidade Federal de Minas Gerais (UFMG), MG, Brazil) spoke about “Virus and host factors associated to SARS-CoV-2 transmission and severe cases of COVID-19”.

Roundtable 2 was exclusively focused on Dengue vaccine and had Dr. Flavio da Fonseca (UFMG) as chair. In this roundtable, the researchers Felipe Lorenzato, Mayuri Sharma and Hansi Dean, from Takeda company (Tokyo, Japan), talked about “Detailed characterization of immune responses to a live-attenuated tetravalent dengue vaccine”.

Finally, for the first time SBV promoted a unique roundtable (roundtable 3) called Young Women Inspiring Research, for which we invited young women researchers to talk about their careers, from students to independent researchers, and to give a lecture about their recent scientific accomplishments and data. Dra. Iranaia Assunção Miranda (Universidade Federal do Rio de Janeiro—UFRJ) conducted the table. The first talk was presented by Dra. Jaqueline Goes de Jesus (Instituto de Medicina Tropical de São Paulo, SP, Brazil) and addressed the “Genomic surveillance of emerging viruses in Brazil”. Dra. Carolina G. de O. Lucas (Yale University School of Medicine, New Haven, CT, United States) presented recent advances regarding “Kinetics of antibody responses dictate COVID-19 disease outcome”.

### 2.5. Abstract and Oral Presentations and the Helio Gelli Pereira Award

In 2020, 369 abstracts were submitted to SBV and accepted for divulgation in the society webpage. The reviewed abstracts represented unpublished data, distributed as basic virology (62, 17%), human and public health virology (177, 48%), environmental virology (43, 12%), veterinary virology (61, 17%), and plant and invertebrate virology (26, 7%). Among those, 30 studies were selected to be presented as short oral presentations. Six oral presentation sections were hosted in the meeting, including two sections of human virology (with nine students total), one of basic virology (with five students), one of veterinary virology (with five students), one of plant and invertebrate virology (with six students), and one of environmental virology (with five students) (Table 2).

Undergraduate, graduate and recently graduated students were also encouraged to apply for the HGP award, which is conferred to complete scientific articles developed by one undergraduate (one award) and one graduate student (one award). All the submitted articles were previously selected by a committee and seven were selected for oral presentation during the congress, with two in the undergraduate and five in the graduate category (Table 3).

The HGP award in the graduate-student category was given to Ricardo S. Cardoso, from FMRP/USP, SP, who presented the work “Host Retromer Protein Sorting Nexin 2 Interacts with Human Respiratory Syncytial Virus Structural Proteins and is Required for Efficient Viral Production” [33]. The undergraduate-category student awarded was Victória Fulgêncio Queiroz, from UFMG, who presented the work “In-depth analysis of the replication cycle and particle structure of Orpheovirus brasiliensis” [34]. The award was supported by SBV, American Society for Microbiology (ASM) and *Viruses*. ASM granted a one-year membership and one eBook to the awardees. *Viruses* offered a full discount for publication by both research groups awarded if the manuscript is accepted by referees and editors. These partnerships between SBV and *Viruses* have been very productive, and one of the works that received the HGP award was recently published at *Viruses* [35], similar to what happen in the last year.

**Table 3:** Oral presentations on the HGP award.

Helio Gelli Pereira Award	
**Undergraduate category**	
Ellen Viana Souza, IAL	Diversity of enteric and non-enteric human adenovirus strains in Brazil, 2006–2011 [36]
Victória Fulgêncio Queiroz, UFMG *	**In-depth analysis of the replication cycle and particle structure of Orpheovirus brasiliensis [34]**
Graduate category	
Bruna Pinheiro-Lima, EMBRAPA	Transmission of the Bean-Associated Cytorhabdovirus by the Whitefly Bemisia tabaci MEAM1 [37]
**Flávia F. Bagno, UFMG ^#^**	**Fast-track development and validation of an enzyme-linked immunoassay kit for diagnosis and surveillance of COVID-19 [38]**
Leonardo Cecílio da Rocha, FAMERP	Enteric viruses circulating in undiagnosed central nervous system infections at tertiary hospital in São José do Rio Preto, São Paulo, Brazil [39]
Paulo V. M. Boratto, UFMG	Yaravirus: A novel 80-nm virus infecting Acanthamoeba castellanii [40]
Ricardo S. Cardoso, USP *	**Host Retromer Protein Sorting Nexin 2 Interacts with Human Respiratory Syncytial Virus Structural Proteins and is Required for Efficient Viral Production [33]**

* The HGP award winning work is highlighted in the table. ^#^ The work received an honorable mention as a highlight during the presentation of the HGP award.

## 3. Conclusions and Remarks

The 31st Brazilian Society for Virology Meeting represented a big but fortunate challenge to the directory board, scientific committee and society members, given the restrictions imposed by the COVID-19 pandemic and the necessity to host an event completely online. Remarkably, the 2020 meeting had a record number of participants, with an extraordinary involvement of students from all over the country. Seminal lectures comprising different virology fields attracted a large audience, which established great discussion in the platforms during all the meetings. The COVID-19 roundtable presented by prominent Brazilian virologists and the Young Women Inspiring Research section were novel and very enthusiastic roundtables, which expressed the SBV aspiration to be even more representative for these members’ community.

Therefore, the 31st SBV annual meeting provided an important opportunity for senior scientists, young researchers and students from Brazil and other countries to share their experience, establish new and consolidated collaborations, and further expand the current knowledge in virology.

## Figures and Tables

**Table 1:** Scientific programing schedule of the 31st Brazilian Society for Virology Congress and 15th Mercosur Virology Meeting.

Day	Activities	Themes
**23 November**		
Monday	Opening Session	Welcome to the participants
	Conference 1—Human Virology	The landscape of host responses and disease pathology in SARS-CoV-2 infection
	Oral presentation 1—	Basic Virology oral presentation of students
**24 November**		
Tuesday	Conference 2—Environmental Virology	Environmental surveillance of enterovirus in support of global eradication activities
	Illumina Conference	The role of new generation sequencing (NGS) in combating COVID-19
	Oral presentation 2	Human Virology oral presentation of students
**25 November**		
Wednesday	Conference 3—Invertebrates and Plants Virology	Ecological complexity, host range evolution, and virus emergence
	Oral presentation 3	Veterinary Virology oral presentation of students
	Roundtable 1	COVID-19 in Brazil
**26 November**		
Thursday	Conference 4—Basic Virology	Targeting immunometabolism to understand COVID-19 disease
	Oral presentation 4	Environmental Virology oral presentation of students
	Oral presentation 5	Invertebrates and Plants Virology oral presentation of students
**27 November**		
Friday	Conference 5—Veterinary Virology	The human-animal interface of SARS-CoV-2
	Oral presentation 6	Human Virology oral presentation of students
	Hélio Gelli Pereira—Award session	Oral presentation of selected studies in different virology area
**28 November**		
Saturday	Roundtable 2	Dengue vaccine
	Conference 6—QIAGEN	Unraveling the SARS-CoV-2 epidemiology by new generation sequencing (NGS)
	Roundtable 3	Young Women Inspiring Research
	Awards announcement	Announcement of the best oral presentations and HGP award winners
	SBV General Assembly	Closing of the 31o SBV meeting

**Table 2:** Oral presentations at the 31st Brazilian Society for Virology Congress and 15th Mercosur Virology Meeting.

First Author and Institution	Title
**Basic Virology**	
Igor de Andrade Santos, UFU	Protein Isolated from Crotalus Durissus Terrificus Strongly Impairs Chikungunya Virus Cycle [3]
Fernando Luz de Castro, UFRJ	Modulation of HERV expression and genes nearby driven by four different arboviruses during infection of human primary astrocytes [4]
Isabella Aquino, UFMG *	**Trapping the Enemy: Vermamoeba Vermiformis Circumvents Faustovirus Mariensis Dissemination by Enclosing Viral Progeny Inside Cysts [5]**
Jonas Nascimento Conde, SBU	Zikv Ns5 Sumoylation Site Regulates Interferon Responses in Primary Human Brain Endothelial Cells [6]
Karine Lourenço, UFMG	Zoonotic Vaccinia Virus Belonging to Different Genetic Clades Present Immunomodulation Abilities That Are Proportional to Their Pathogenic Potential [7]
**Human Virology 1**	
Alice Soares, UCSAL	Humoral Immunity Monitoring in Patients Infected with Sars-Cov-2, in Salvador, Bahia, Brazil [8]
Rosa Maria Mendes Viana, USP	Detection of Respiratory Virus in Primary Cholesteatoma [9]
Giovana Santos Caleiro, IMTSP	Detection of Saint Louis Encephalitis Virus (Slev) in Aedes Aegypti (Diptera: Culicidae), Araçatuba, Brazil [10]
Joseane Mayara Almeida Carvalho, UNIFESP	Dynamics of Sars-Cov-2 Transmission in Families of Patients and Healthcare Workers from Two Hospitals in São Paulo City [11]
**Veterinary Virology**	
Raíssa Canova, UPF	Bovine Leukemia Virus from Cattle and Human Are Genetically Related [12]
Amanda Gonzalez da Silva, UFRGS *	**Fta® Sampling Cards as A Tool for Surveillance and Diagnosis Support** of **Rabies Virus** in **Animal Sample [13]**
Matheus Nunes Weber, FEEVALE	Positive Serology for Saint Louis Encephalitis Virus and West Nile Virus in Horses from Rio Grande Do Sul [14]
Ruy Diego Chacón Villanueva, USP	Molecular Characterization of Marek´s Disease Virus Strains Associated with Outbreaks in Commercial and Backyard Poultry in Brazil [15]
Laura Morais Nascimento Silva, UFV	Brazilian Commercial Vaccines Against Cpv-2 Genetically Differ from Field Strains in The Antigenic Protein Vp2 [16]
**Environmental Virology**	
Gustavo Farias, FIOCRUZ	Circulation of Orthohantavirus in Rodents from Bahia State, Northeast Region of Brazil [17]
Macyclelma Alves Albuquerque, IEC	Detection of Rotavirus A (Rva) in Oyster-Farming and Waters Before and After Purification Process in A Cit in The State of Pará [18]
Déborah Carolina Carvalho dos Anjos, UFG *	**Evaluation** of **The Presence and Quantification** of **Norovirus and Sapovirus Load** in **Samples Collected** in **Sewage Treatment Stations** in **The Federal District, Brazil [19]**
Paula Rogovski, UFSC	Detection and Metagenomic of Sars-Cov-2 in Human Sewage in Santa Catarina, Brazil, November 2019 [20]
Isabela da Silva Paes, UFV	Heterological Expression of Virion-Associated Peptidoglycan Hydrolases of Phage Vb_Ecom-Ufv13 [21]
**Invertebrates and Plants Virology**	
Luana Lucas Dutra, UFV *	**The Effects** of **Temperature on The Honey Bees’ Immune Response Against Acute Bee Paralysis Virus [22]**
Hugo De Paula Oliveira, UFSM	Genomic Characterization of An Alphabaculovirus Infectious to The Cotton Armyworm, Alabama Argillacea [23]
Osvaldo Franco De Araújo, UFT	Prevalence of Newly Characterized Phasivirus in Mosquitoes from Tocantins State, Brazil [24]
Camila Chabi De Jesus, USP	Evolutionary Reconstruction of Citrus Leprosis Virus C Population Suggests Its Diversification into Three Lineages Before the Introduction of Citrus Spp. in America [25]
Ricardo José Gonzaga Pimenta, UNICAMP	Genome-Wide Investigation of Resistance to Sugarcane Yellow Leaf Virus by Machine Learning Approaches [26]
João Paulo Da Silva, UFV	Evolution of A Dna Plant Virus in The Natural Environment - A Journey Through Time [27]
**Human Virology 2**	
Karen Steponavicius Cruz Borbely, UFAL	Term Placental Extravillous Cytotrophoblast Cells Are Infected by Zika Virus [28]
Rodrigo Lopes Sanz Duro, USP	Analysis of Gut Viroma in Patients Newly Diagnosed with Human Immunodeficiency Virus (HIV) [29]
Felipe Rinald Barbosa Lorenzato, CAIMED	Efficacy of A Tetravalent Dengue Vaccine in Healthy Children and Adolescents [30]
Karolina Lopes Dias, UFMG	Gene Expression of Components of The Rap1 Pathway and Their Micrornas in Organotypic Cultures of Human Keratinocytes Expressing Hpv16 E6 And E7 Oncoproteins [31]
Emily Montosa Nunes, ICESP *	**Two Natural Human Papillomavirus 18 Variants Exhibit Functional Differences** in **Human Keratinocytes [32]**

* The best presentation is highlighted in the table.

## Data Availability

This study did not report any data.
